# ZNF300 tight self-regulation and functioning through DNA methylation and histone acetylation

**DOI:** 10.1186/s13578-017-0160-8

**Published:** 2017-06-28

**Authors:** Feng-Juan Yan, Jingyi Fan, Zan Huang, Jun-Jian Zhang

**Affiliations:** 1 College of Life Science, Wuhan University, Wuhan, 430072 Hubei People’s Republic of China; 2 Department of Pediatrics, Zhongnan Hospital of Wuhan University, Wuhan, 430072 Hubei People’s Republic of China; 3 Department of Neurology, Zhongnan Hospital of Wuhan University, Wuhan, 430072 Hubei People’s Republic of China

**Keywords:** KRAB-ZFP, ZNF300, DNA methylation, Histone acetylation

## Abstract

**Background:**

Accumulating evidence demonstrates that the KRAB-ZNFs involve in various biological processes. As a typical member of KRAB-ZNFs, dysregulation of ZNF300 contributes to multiple pathologies such as leukemia and cancer. However, mechanisms underlying ZNF300 tight regulation and its pathophysiological function remain largely unknown.

**Methods:**

The effect of ZNF300ZFR on gene transcriptional activity was measured by Dual luciferase reporter system. ChIP-PCR assay were performed to detect the enrichment of ZNF300 protein and H3K9Ac in the ZNF300 gene. Co-immunoprecipitation assays followed by western blot were performed to detect the interaction between ZNF300 and KAP1. The DNA methylation in the ZNF300 gene promoter was analyzed by BSP. ZNF300 function on K562 cell differentiation was analyzed by flow cytometry.

**Results:**

In this study, we found that the zinc finger domain-encoding region (ZFR) of ZNF300 functioned as a repressor possibly by mediating DNA methylation and ZNF300 bound to its ZNF300ZFR, suggesting a potential auto-inhibition mechanism. To support this, DNA methylation inhibition upregulated ZNF300 expression and ZNF300 overexpression inhibited endogenous ZNF300 expression. More importantly, DNA methylation inhibition restored megakaryocyte differentiation in K562 cells suppressed by ZNF300 downregulation, suggesting an important role of DNA methylation in ZNF300 function. Interestingly, ZNF300 knockdown restored global H3K9Ac that was reduced in K562 cells undergoing megakaryocyte differentiation.

**Conclusions:**

Our study revealed novel features of ZNF300 that possibly mediate its regulation and function by modulating epigenetic modifications.

## Background

Krüppel-associated box-containing zinc finger proteins (KRAB-ZFPs) belong to the largest gene family of transcription factors in eukaryotes [1], which makes up approximately one-third of the zinc finger proteins identified in human genome. Typical KRAB-ZFPs contain KRAB domain and zinc finger domain linked by a spacer region. The KRAB domain consists of one or both of A box and B box [2], of which the A-box is highly conserved and plays a key role while the B-box is less conserved and plays an auxiliary role [3]. The KRAB domain acts as a transcriptional repressor by recruiting corepressor proteins such as KAP1, HP1, HDAC and Setdb1 [4–7] and induces heterochromatin formation through DNA methylation, histone modifications [8–10], and nucleosome remodeling [11]. The zinc finger domain of KRAB-ZNFs contains 4–30 zinc finger motifs (C_2_H_2_ type) and binds to DNA. In addition, two adjacent zinc finger motifs are usually connected by a highly conserved sequence “TGEKPYX” [12].

Accumulating evidence demonstrates that different members of the KRAB-ZNFs involve in various biological processes such as stem cell biology [13], cell cycle regulation [14], methylation of imprinted genes [15], suppression of endogenous retroviruses [16], and spermatogenesis [17]. Dysregulation of KRAB-ZFPs has been indicated in multiple pathological processes including tumor and leukemia formation. For instance, ZNF268b2 expression is associated with cervical cancer [18] while ZNF300 expression correlates to blood cell maturation and leukemogenesis possibly by affecting terminal differentiation of blood cells [19]. As a typical member of KRAB-ZNFs, ZNF300 encodes a KRAB domain and zinc finger domain with 12 zinc finger motifs of C2H2 type, which binds to the consensus sequence C(t/a)GGGGG(g/c)G. The ZNF300-binding sites have been found in the promoter regions of multiple genes such as IL-2, IL2RB, CD44, TP53, TNFα, TRAF2 [20]. In addition, ZNF300 has been shown to act as a signaling molecule to enhance NF-κB signaling and promote cervical cancer cell proliferation [21]. Nevertheless, ZNF300 target genes have not been well characterized and how ZNF300 controls target genes remains unclear.

In this study, we discovered that the last exons of KRAB-ZFPs encoding the zinc finger domain mediated auto-inhibition of gene transcription. As a model, ZNF300 bound to its zinc finger domain-encoding region and induced DNA methylation. ZNF300 also altered histone 3 lysine 9 acetylation in leukemic cells that was forced to undergo terminal differentiation. Furthermore, interfering epigenetic modifications overwhelmed the effect of ZNF300 depletion. Our study reveals novel features of ZNF300 regulation and function. These findings help us understand the nature of KRAB-ZFPs.

## Methods

### Cell culture and compound treatment

HEK (human embryonic kidney)-293T cells were maintained in a complete Dulbecco’s modified Eagle’s medium and K562 was cultured in a complete RPMI 1640 medium (Gibco BRL, Grand Island, NY, USA) both of which were supplemented with 10% fetal bovine serum and penicillin/streptomycin and were cultured in a humidified chamber with 5% CO_2_ atmosphere at 37 °C. For compound treatment, 5-aza-2′-deoxycytidine (5-AzadC, 5 μM), and 12-*O*-tetradecanoylphorbol 13-acetate (PMA, 10 nM) were used.

### Retroviral and lentiviral transduction

For enforced expression of ZNF300, lentivirus system (pHAGE vector) was used. Lentivirus packaging and infection were performed as previously described [22, 23]. All vectors carried puromycin-resistant gene and the transduced cells were selected with puromycin (2 μg/mL) for a week to obtain stable cell lines. ZNF300 was fused with a Flag tag in the C-terminal. Empty plasmids were used as control vector.

### Dual luciferase activity assay

All luciferase reporter constructs expressed firefly luciferase and assays were performed in 293T cells. Briefly, 3.0 × 10^4^ 293T cells were seeded (48-well plate) the day before transfection. When cells reached to 70% confluence, firefly luciferase reporter plasmid in combination with other plasmids as indicated in the figures were used for transfection. Same amount of pRL-TK expressing Renilla luciferase was used in all samples and served as internal control. The total amount of plasmids in each transfection was kept constant by using empty vector where required. Cells were lysed 24 h post-transfection with Passive Lysis Buffer and the dual luciferase activity was assayed by the dual-luciferase reporter assay according to the manufacturer’s instructions (Dual-Luciferase Reporter Assay System, Promega, Madison, WI, USA). The firefly luciferase activity was normalized to the Renilla luciferase activity and presented as relative luciferase activity. Values were mean ± SD from three independent experiments. All of the transfection assays were performed with transfection reagent polyethyleneimine “MAX” (Polysciences Inc).

### RNA isolation and quantitative RT-PCR

Total RNA was extracted from cells using TRIzol reagent (Invitrogen, Grand Island, NY, USA) according to the manufacturer’s instructions. cDNA was synthesized by M-MLV (Moloney murine leukaemia virus) reverse transcriptase (Invitrogen, Grand Island, NY, USA) from 2 μg of total RNA. Quantitative PCR was carried out under the following conditions: 95 °C for 15 min followed by of 95 °C for 30 s, 63 °C for 30 s and 72 °C for 30 s for 40 cycles. The reactions were performed using the Power SYBR Green PCR Master Mix with triplicates on the ABI7500 real-time PCR System (Applied Biosystems). For each primer set, the C_t_ value was normalized to that of *GAPDH* (glyceraldehyde-3-phosphate dehydrogenase) as inner control, which was further normalized to that of control sample. The relative quantitation of PCR product was measured using the comparative ΔΔC_t_ method and presented as relative mRNA level. Primer sequences are available upon requested.

### Plasmid construction

The DNA sequences of zinc finger domain-encoding region of ZNF300, ZNF268, ZNF446, GATA1 or truncated forms of the zinc finger domain-encoding region of ZNF300 were amplified by PCR and cloned into pGL3-promoter vector between *Bam*HI and *Sal*I sites. The CMV, LTR, and *ZNF300* gene promoter (−1900 to +150 relative to the transcription start site) were amplified by PCR and subcloned into pGL3-promoter vector between *Xho*I and *Hin*dIII sites to replaced SV40 promoter. To construct gRNA expressing vectors, the gRNA sequences targeting zinc finger domain-encoding region of ZNF300 were determined by online gRNA searching tool (http://crispr.mit.edu/) and blasted at NCBI to avoid off-target. The sequences of gRNA for human ZNF300 are listed as following: gRNA#1, GAATTCGCTGGTGTCCCGGA; gRNA#2, GCCGTATGAGTGTACCGAATG; gRNA#3, GCCCGCATTCACTACATTCAT; gRNA#4, GCCTATGAATGTAGAGAGTGT; gRNA#5, GAGTTGTGACTTCTTAGCAA; gRNA#6, GTACAGTTAGTTGTGACTTC. Two complementary oligonucleotide strands annealed to form a double strand structure and subcloned into the pGL3-U6-gRNA-puromycin vector.

### Methylation-specific PCR (MSP) and bisulfite sequencing

293T cells were transfected with pGL3-ZNF300pro-luc plasmids with or without zinc finger domain-encoding region of ZNF300 on the downstream. The plasmid DNA was extracted from the transfected cells according to the manufacturer’s instructions and treated with sodium bisulfite using the EZ DNA Methylation-Gold Kit™ following the manufacturer’s guidelines. One to two ng of sodium bisulfite-converted plasmid DNA was used as a template for methylation specific PCR (MSP). For MSP we designed primer pairs for ZNF300 gene promoter. Primers for DNA analysis were designed using the Methprimer Software (http://www.urogene.org/methprimer/). PCR was conducted using a pfu DNA polymerase (Thermo Scientific), with an initial denaturation step at 95 °C for 10 min, followed by 35 cycles of denaturation at 95 °C for 30 s, annealing at the respective Tm for each set of primers for 30 s, and extension at 72 °C for 1 min. PCR amplicons were purified with a PCR purification Kit. The PCR fragments were ligated into pGEM-T Easy vector (Promega, Madison, WI, USA). Cloned plasmids were transformed into DH5α competent cells. Transformed cells were selected using LB/ampicillin agar plates. Colonies were randomly picked to extract plasmid DNA for sequencing. During bisulfite conversion, cytosines (C) are converted into thymidines (T), but 5-methylcytosines remain unaltered. DNAMan program was used for sequence alignment and analysis.

### Chromatin immunoprecipitation assay

ChIP assays were performed by using antibodies specific for Flag tag (Cat# F3165, Sigma), ZNF300 (Cat#SAB2102853, Sigma), acetylated histone 3 lysine 9 (Cat# 9649,CST), or a normal mouse/rabbit IgG as previously described [22]. The chromatin DNA enrichment of the zinc finger domain-encoding region and promoter regions of *ZNF300* gene, or the control region of *GAPDH* gene promoter was determined by quantitative PCR. The relative occupancy was calculated by 2^(C^_t_^_IP − C^_t_^_Input)^. *GAPDH* promoter served as negative control region. Primer sequences are available upon requested.

### Co-immunoprecipitation assay

The ZNF300 and KAP1 gene were cloned into a vector containing a Flag or HA tag. The plasmids were transiently transfected into the 293T cells. After 24 h, the transfected cells were lysed as previous described [23]. Cell lysates were incubated with the appropriate monoclonal antibody Flag, as well as 30 μL of a GammaBind Plus-Sepharose (GE Healthcare, Logan, UT, USA). After an overnight incubation at 4 °C, the Sepharose beads were washed five times with 1 mL of lysis buffer. The immunoprecipitates were fractionated by SDS-PAGE, and western blot analysis was performed. All the immunoprecipitation experiments were repeated three times, and similar data were obtained.

### Statistical analysis

Data combined from three or more independent experiments are given as the mean ± STDEV. All statistical analyses were performed using the *Student’s t test* (two-tailed, unpaired). A p value of 0.05 or less was considered significance.

## Results

### Zinc finger domain-encoding regions of KRAB-ZFPs mediate gene suppression

**Fig. 1:**
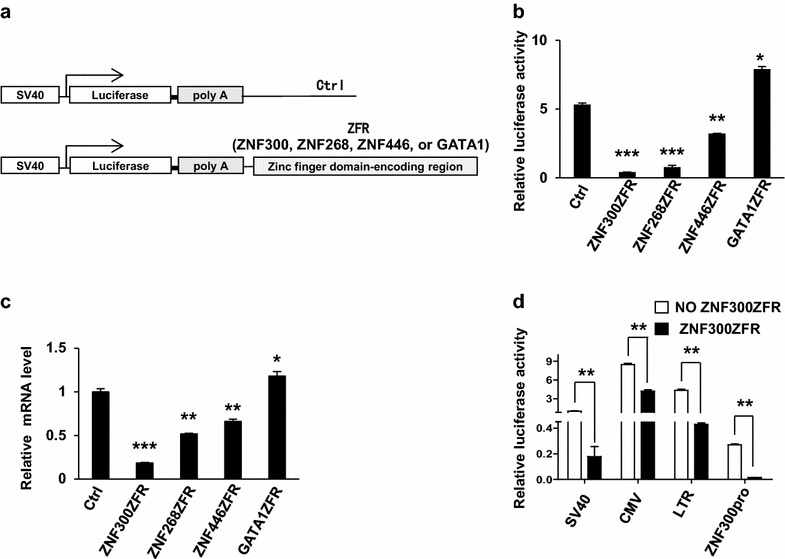
Zinc finger domain-encoding regions of KRAB-ZFPs mediate gene suppression. **a** Schematic illustration of reporter vectors shows SV40 promoter, the firefly luciferase, polyA element, and zinc finger domain-encoding regions (ZFR) of different zinc finger proteins. **b** 293T cells were transfected with firefly luciferase reporter vectors pGL3-promoter plasmids with different insertions as indicated. Luciferase activities were measured and presented as relative luciferase activity. **c** The mRNA level of firefly luciferase in the transfected 293T cells was measured by quantitative RT-PCR and represented as relative mRNA level. **d** 293T cells were transfected with luciferase reporter vectors driven by different promoters with (ZNF300ZFR) or without (NO ZNF300ZFR) zinc finger domain-encoding region of *ZNF300* gene on the downstream as indicated. The luciferase activities were measured, normalized, and presented as relative luciferase activity. *, **, and *** indicate significance compared to control (p < 0.05, p < 0.01, and p < 0.001, respectively). All values are statistics of three independent experiments with triplicates and presented as mean ± STDEV

We previously studied the function of ZNF268 and ZNF300 [7, 18, 19, 24]. It was found that these full-length proteins were very difficult to be overexpressed as reported in other studies [25]. We speculated that the zinc finger domain-encoding regions (ZFR) of KRAB-ZFPs might mediate inhibitory effect on transcription. To test this, a luciferase reporter system was developed. The ZFR encoding the zinc finger domains of ZFPs were subcloned into the downstream of the poly A element in luciferase reporter vector (Fig. 1a). Thus the ZFR would not be transcribed or translated, which excluded any possible regulation at post-transcriptional level. We cloned the ZFRs of ZNF300, ZNF268, and ZNF446 into the report system (abbreviated as ZNF300ZFR, ZNF268ZFR, and ZNF446ZFR, respectively), which contains 12, 24, or 3 zinc finger motifs, respectively. As shown in Fig. 1b, ZNF300ZFR, ZNF268ZFR, and ZNF446ZFR significantly reduced the luciferase activities. As a control, the ZFR from GATA1 (GATA1ZFR), which is not a member of KRAB-ZFPs, did not show any inhibitory effect. Consistent to luciferase assay, ZNF300ZFR, ZNF268ZFR, and ZNF446ZFR downregulated the mRNA level of firefly luciferase gene proportional to luciferase protein activity reduction while GATA1ZFR did not (Fig. 1c). These results suggest that the ZFRs of KRAB-ZNFs encoding zinc finger domain of these genes may act as a cis-element to mediate gene suppression.

To exclude that the inhibitory effect of ZFRs may be specific for SV40 promoter, ZNF300ZFR was used as a model for further experiments and the SV40 was replaced with CMV promoter, LTR (long terminal repeat from HIV-1), or *ZNF300* gene promoter (ZNF300pro, nucleotides −1900 to +150 relative to the transcription start site). Without ZNF300ZFR (NO ZNF300ZFR), these promoters all nicely drove luciferase expression (Fig. 1d). Once ZNF300ZFR was placed on the downstream, the luciferase activities were significantly reduced (Fig. 1d). These results suggest that the inhibitory effect of ZFRs is universal for different promoters.

### Collaboration of multiple zinc finger motifs is required for optimal inhibition function of ZNF300ZFR

**Fig. 2:**
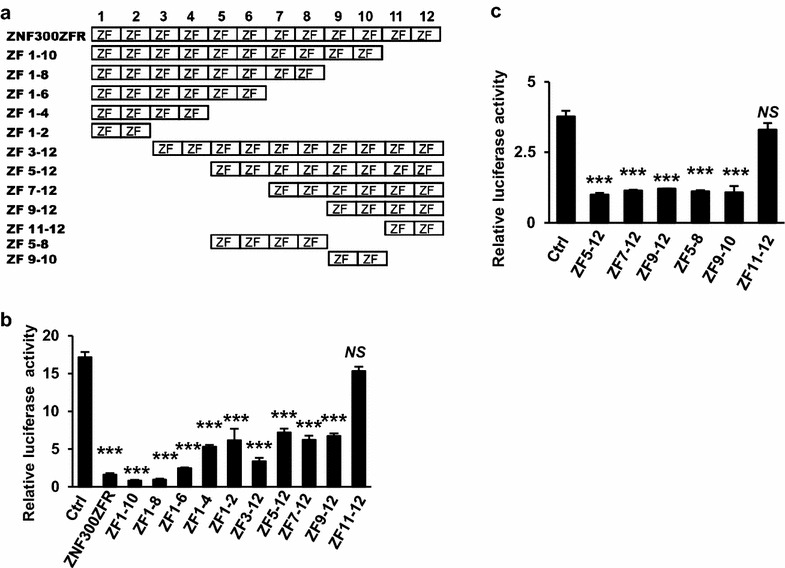
Collaboration of multiple zinc finger motif-encoding regions is required for optimal inhibition function of ZNF300ZFR. **a** Schematic representation of zinc finger motif-encoding regions (ZF) in the ZNF300ZFR as well as different truncated forms. The *number on the top* represents the position of the zinc finger motif-encoding regions in ZFR of ZNF300. **b**, **c** 293T cells were transfected with pGL3-promoter plasmids with insertion of different ZNF300ZFR truncations. The luciferase activity was measured, normalized, and presented as relative luciferase activity. *** indicates significance compared to control (p < 0.001). *NS* non-significance. All values are statistics of three independent experiments with triplicates and presented as mean ± STDEV

To further map the precise regions within ZNF300ZFR that were responsible for transcription repression, a serial truncation forms of ZNF300ZFR were constructed and subcloned into the luciferase reporter vector as described above. Each time, two DNA encoding zinc finger motif from the 3′ of ZFR was deleted (Fig. 2a). For instance, ZF1-10 represents the truncated ZFR spanning from the 1st to the 10th zinc finger motif-encoding regions of ZNF300ZFR and so on. As shown in Fig. 2b, c, the inhibitory activity of ZF1-10 and ZF1-8 was comparable to that of full length ZNF300ZFR whereas ZF1-6 started to loose inhibitory activity and a significant proportion of inhibitory activity was lost for ZF1-4 and ZF1-2, suggesting a critical role of zinc finger motif-encoding regions from the 5th to 8th zinc finger motif (ZF5-8). Consistent with intact inhibitory function of ZF1-10, ZF11-12 completely lost the suppression ability. In contrast, truncations deleted from 5′ including ZF5-12, ZF7-12, and ZF9-12 retained similar inhibition ability comparable to that of ZF1-2 and ZF1-4, suggesting regions from the 9th to 10th zinc finger motif-encoding region (ZF9-10) may be important. Similarly, significant loss of inhibition ability of ZF3-12 compared to that of ZNF300ZFR suggested an important role of zinc finger motif-encoding regions from the 1st to 2nd zinc finger motif (ZF1-2). However, further test showed that ZF1-2, ZF5-8, and ZF9-10 possessed similar inhibition ability to that of ZF5-12, ZF7-12, and ZF9-12. Altogether, these observations suggest that collaboration of multiple zinc finger motif-encoding regions is required for optimal inhibition function of ZNF300ZFR.

### The ZNF300 protein directly binds to ZNF300ZFR

**Fig. 3:**
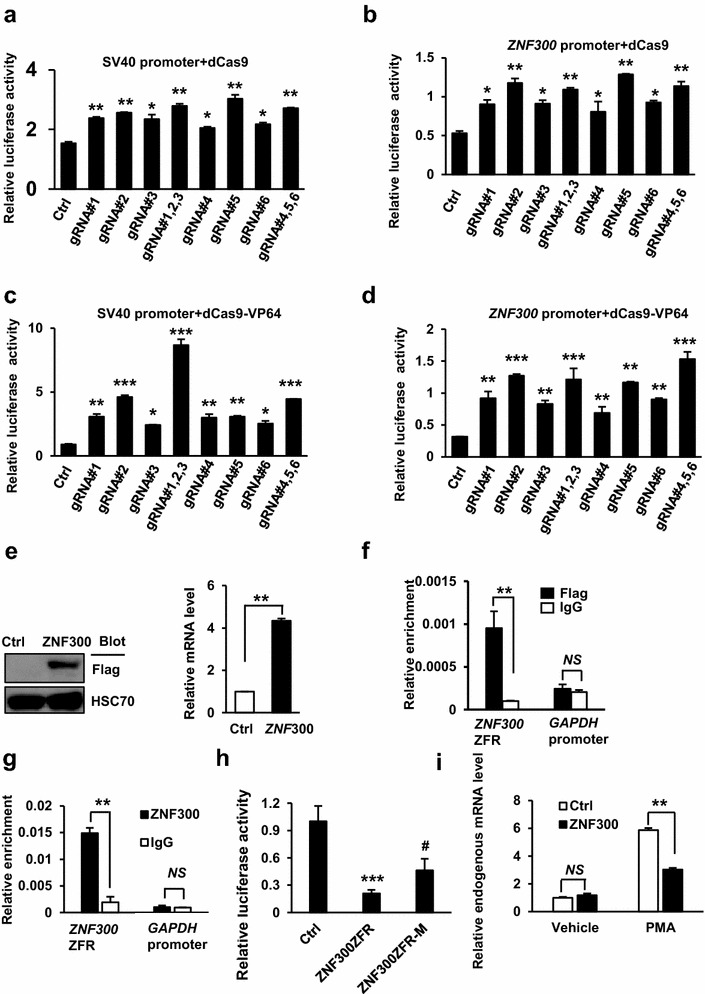
The ZNF300 protein directly binds to ZNF300ZFR. **a**, **b** 293T cells were co-transfected with firefly luciferase reporter vector driven by SV40 promoter (**a**) or *ZNF300* promoter (**b**) with ZNF300ZFR on the downstream of polyA element as described in Fig. 1d, inner control renilla luciferase reporter vector, dCas9 expressing vector, and in combination with different gRNAs specific for ZFR of ZNF300 as indicated. The promoter activity was measured, normalized, and presented as relative luciferase activity. **c**, **d** Same experiment procedure as described in **a**, **b** except that the dCas9 expressing vector was replaced with a vector expressing dCas9 fused with VP64. *, **, and *** indicate significance compared to control (p < 0.05, p < 0.01, and p < 0.001, respectively). Data (mean ± STDEV) are statistics of one representative results (triplicates) from three independent experiments with similar results. **e** K562 cells were transduced with control lentiviral vector (Ctrl) or a lentiviral vector expressing ZNF300. The exogenous ZNF300 protein in the resultant cells was detected by Western blot using antibody specific to Flag tag fused to exogenous ZNF300 (*left panel*). HSC70 serves as a loading control. The relative expression level of ZNF300 mRNA in the resultant cells was measured using quantitative RT-PCR (*right panel*). **f** K562 cells overexpressing Flag-ZNF300 were cross-linked and used for ChIP assay with Flag antibody or normal mouse IgG. The DNA amount of ZNF300ZFR and *GAPDH* promoter region was measured by quantitative PCR. The DNA amount was normalized to that of 1% input DNA and presented as relative enrichment. **g** ChIP-PCR was performed in K562 cells using antibody recognizing the endogenous ZNF300. **h** Wild-type (ZNF300ZFR) or mutant ZNF300ZFR (ZNF300ZFR-M) were subcloned into luciferase activity reporter vector driven by ZNF300 promoter as described in Fig. 1. **i** K562 cells overexpressing ZNF300 and control cells were treated with vehicle or PMA. The endogenous ZNF300 mRNA expression were measured by quantitative RT-PCR using a pair of primers recognizing the 3′ UTR that was absent in the exogenous ZNF300 expressing vector

Based on our observations, we hypothesized that ZFR recruited inhibitory machinery to suppress gene transcription. Therefore, blocking the inhibitory machinery binding to the ZFR would ameliorate its inhibition. To achieve this, we took advantage of CRISPR/Cas9 technique. Recent studies showed that recruitment of the nuclease-deficient Cas9 (dCas9 with D10A and H840A mutations) to the interested gene loci directed by sequence-specific guide RNAs (gRNAs) potently regulated gene expression through sterically competing with potential transcription regulatory factors [26]. In addition, dCas9 may exert enhanced ability to inhibit or activate gene expression by fusing with KRAB or VP64 (suppression or activation domain, respectively) [26, 27]. To test this possibility, six pairs of gRNAs specific for different regions of ZNF300ZFR were designed. Firefly luciferase reporter vectors with ZFR on the downstream as described in Fig. 1d and dCas9 expressing vector in recombination with vectors expressing various gRNAs were co-transfected into 293T cells. As expected, the luciferase activity driven by SV40 or *ZNF300* gene promoter was significantly increased by gRNAs (around twofolds increase) compared to control (Fig. 3a, b). Notably, the effect was more dramatic when dCas9-VP64 (dCas9 fused with the activation domain VP64) was overexpressed (Fig. 3c, d). These results demonstrate that dCas9 recruitment to the ZNF300ZFR directed by gRNAs effectively antagonizes the inhibitory effect of ZNF300ZFR. These observations support our idea that inhibitory machinery may be recruited to the ZNF300ZFR.

A recent KAP1 ChIP-Seq study showed significant enrichment of KAP1 on the 3′ end of the KRAB-ZFP genes including ZNF300 [28]. Since KAP1 is a primary partner of KRAB-ZFPs, KRAB-ZFPs might form complex with KAP1 and bind to ZFR to mediate gene suppression. Interestingly, sequence analysis revealed two putative ZNF300-binding sites [consensus sequence C(t/a)GGGGG(g/c)G] between the second and the forth zinc finger motif-encoding region in ZNF300ZFR [20]. Thus, we speculated that ZNF300 might bind to its own ZFR and mediate the inhibitory effect of ZNF300ZFR. To test this, an exogenous Flag-tagged ZNF300 (Fig. 3e) was overexpressed through lentiviral transduction and ChIP-PCR with Flag antibody was performed to detect ZNF300 binding to this putative site. As shown in Fig. 3f, the DNA amount of the putative ZNF300-binding site co-immunoprecipitated by Flag antibody was much more (enriched by approximate ninefolds) than that by normal mouse IgG control antibody. As a negative control, the amount of DNA from *GAPDH* promoter region was comparable in both groups. Moreover, an antibody recognizing the endogenous ZNF300 was also used to perform ChIP-PCR. Again, a significant enrichment in the putative ZNF300-binding site was observed in ZNF300 antibody group compared to normal rabbit IgG control (Fig. 3g). These data demonstrate that ZNF300 is able to bind to its endogenous ZFR locus. To further demonstrate the important role of ZNF300-binding in the inhibitory effect of ZNF300ZFR, the ZNF300-binding sites was mutated (ZNF300ZFR-M) by replacing the core sequence GGGG with ATAT. ZNF300ZFR-M showed reduced ability to suppress the luciferase activity compared to intact ZNF300ZFR (Fig. 3h). Our previous study showed that PMA treatment promoted megakaryocyte differentiation and upregulated ZNF300 expression in K562 cells [19]. Finally, we demonstrated that the upregulation of endogenous ZNF300 mRNA by PMA was reduced by the overexpression of the exogenous ZNF300 (Fig. 3i). Collectively, our data indicate that ZNF300 directly binds to ZNF300ZFR and may mediate the self-regulation of ZNF300 from its endogenous locus.

### ZNF300ZFR mediates DNA methylation

**Fig. 4:**
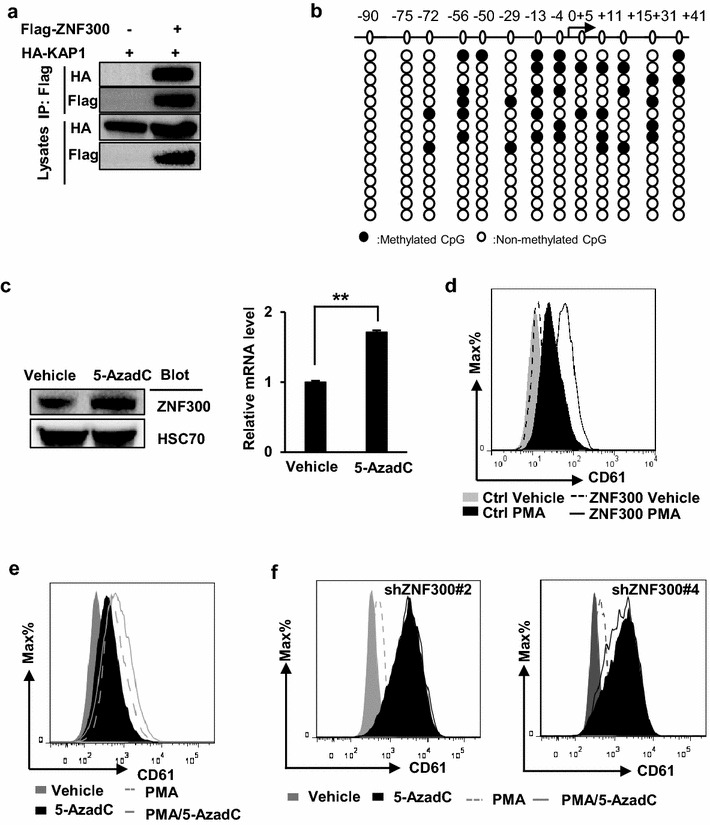
ZNF300ZFR mediates DNA methylation. **a** A co-immunoprecipitation assay was performed in HEK293T cells co-transfected with Flag-ZNF300 and HA-KAP1 to examine the interaction between ZNF300 and KAP1. **b** Schematic illustrations show the position of CpG sites (*circles*). The *arrow* indicates the transcription start position and *numbers* indicate the position of CpG sites relative to transcription start site (*upper*). Bisulfite sequencing analysis the methylated CpGs in the ZNF300 promoter (ZNF300pro) with ZNF300 ZFR on the downstream as described in Fig. 1d. *Black circles* indicate methylated CpGs and *open circles* indicate non-methylated CpGs (*lower*). **c** K562 cells were treated with vehicle or 5-AzadC. The ZNF300 protein was measured by Western blot (*left panel*) and the ZNF300 mRNA expression was measured by quantitative RT-PCR (*right panel*). **d** K562 cells treated with PMA or vehicle as indicated for 2 days. The CD61 expression of the resultant cells was measured by staining cells with anti-CD61-PE antibody and analyzed by flow cytometry. **e** K562 cells were treated with vehicle, PMA, 5-AzadC, or PMA/5-AzadC and the CD61 expression were measured by staining cells with an anti-CD61-PE antibody and analyzed by flow cytometry. **f** ZNF300 knockdown cells (shZNF300 #2 or #4) were treated with PMA/5-AzadC as indicated. The CD61 expression of the resultant cells were measured by staining cells with an anti-CD61-PE antibody and analyzed by flow cytometry

KRAB-ZFPs are known to recruit KAP1 that subsequently mediate heterochromatin formation by inducing long range of epigenetic modification changes including DNA methylation [28–30]. Our experiment confirmed the ability of ZNF300 to recruit KAP1 (Fig. 4a). Therefore, whether ZNF300ZFR mediated DNA methylation was further tested. By using online software MethPrimer program, a CpG island region containing 13 CpG sites between nucleotides −90 to +41 relative to the transcription start site was identified (Fig. 4b). Then the luciferase report vector directed by ZNF300 promoter (ZNF300pro) with or without ZNF300ZFR on the downstream as described in Fig. 1d was transfected into 293T cells. The DNA methylation in the *ZNF300* gene promoter was analyzed by BSP. In total, 15 clones were picked for sequencing in both groups. As control, ZNF300pro without ZNF300ZFR on the downstream did not show any methylated CpG sites (data not shown). In contrast, 9 out of 15 colonies showed methylated CpG sites and 35 methylated CpG sites were identified in total 195 CpGs (17.9% methylated CpG sites) (Fig. 4b). These observations suggest that ZNF300 binding to the ZNF300ZFR may induce DNA methylation and subsequently inhibit *ZNF300* gene expression. To support this idea, a potent demethylating reagent 5-aza-2′-deoxycytidine (5-AzadC, optimal dosage of 5 μM that did not cause obvious cell death in 24 h) increased ZNF300 expression at both mRNA level and protein level (Fig. 4c). It was verified that ZNF300 upregulation enhanced megakaryocyte differentiation induced by PMA (Fig. 4d). Furthermore, 5-AzadC treatment alone promoted megakaryocyte differentiation comparable to that of PMA and enhanced the effect of PMA (Fig. 4e), suggesting an important role of DNA methylation in this biological process. More importantly, 5-AzadC treatment restored megakaryocyte differentiation suppressed by ZNF300 knockdown as previously reported [19] (shZNF300#2 and shZNF300#4) (Fig. 4f). Taken together, our results suggest that ZNF300 tight regulation and functioning may involve DNA methylation.

### The ZNF300 alters histone 3 lysine 9 acetylation

**Fig. 5:**
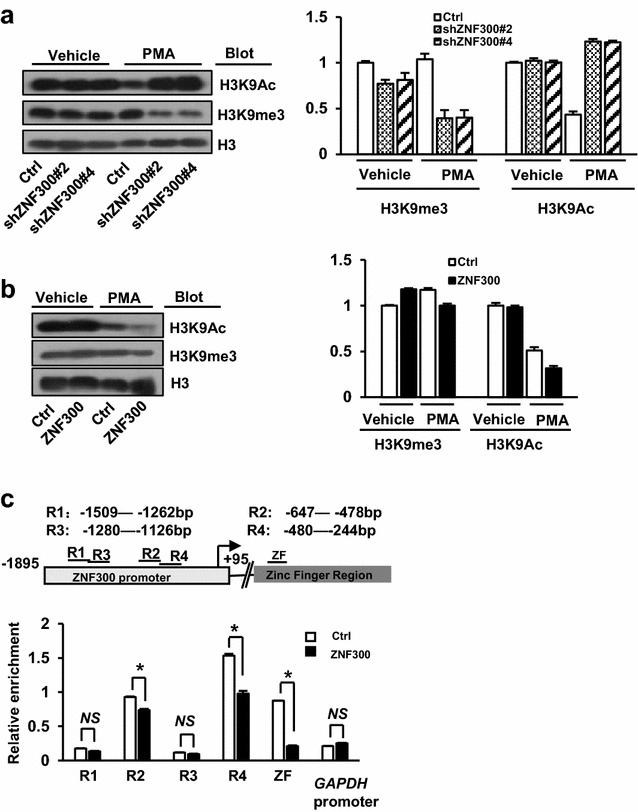
The ZNF300 alters histone 3 lysine 9 acetylation. **a**, **b** Control (Ctrl), ZNF300 knockdown (shZNF300#2 and 4), or ZNF300 overexpression K562 cells were stimulated with vehicle or PMA and harvested for Western blot to measure H3K9 acetylation (H3K9Ac) and 3-methyl-H3K9 (H3K9me3) (*left panel*). Histone3 (H3) serves as loading control. All data are representative blots from two independent experiments with similar results and *right panel* was the average densitometry of the Western blot relative to the control. **c** Control (Ctrl) and ZNF300 overexpression K562 cells treated with PMA were harvested for ChIP assay with anti-H3K9Ac antibody. The DNA amount of *ZNF300* gene promoter (indicated as R1, R2, R3, R4 in the *top panel*), ZNF300 zinc finger domain-encoding region (indicated as ZF in the *top panel*), and *GAPDH* gene promoter was quantified, normalized, and presented as relative enrichment. *GAPDH* serves as a negative control. Top schematic illustration shows the DNA regions used for quantitative PCR. *Numbers* indicate the nucleotide position relative to transcription start site. Data (mean ± STDEV) are statistics of one representative results (duplicates) from two independent experiments with similar results

Heterochromatin formation caused by KRAB-ZNFs also involves HDAC [31]. Therefore, whether ZNF300 functioned to alter histone acetylation was tested by measuring the global histone 3 lysine 9 acetylation (H3K9Ac) and H3K9 tri-methylation (H3K9me3), markers for chromatin in open or close status, respectively. At baseline level, ZNF300 downregulation or overexpression did not change H3K9Ac and H3K9me3 status (Fig. 5a, b). However, PMA-induced megakaryocyte differentiation caused decrease of H3K9Ac whereas ZNF300 knockdown reversed the phenotype (Fig. 5a). Consistently, ZNF300 overexpression showed opposite phenotype: H3K9Ac was further decreased by ZNF300 upon PMA induction (Fig. 5b). In contrast, H3K9me3 was reduced in ZNF300 knockdown cells but no change in ZNF300-overexpressing cells (Fig. 5a, b). Furthermore, the alteration of H3K9Ac in the *ZNF300* promoter region and ZNF300ZFR was confirmed by ChIP-PCR (Fig. 5c). Our results suggest that ZNF300 may alter H3K9Ac.

## Discussion

Dysregulation of ZNF300 contributes to multiple pathologies including leukemia and cancer [19, 21, 24]. Understanding how ZNF300 are tightly regulated and how ZNF300 functions are critical to reveal full picture of ZNF300. In this study, we revealed several novel points regarding ZNF300 function and regulation, which might be common features of KRAB-ZFPs.

Our study revealed an important role of zinc finger domain-encoding regions in regulating the expression of KRAB-ZFP genes. We provided the first line of evidence showing that the zinc finger domain-encoding regions from different KRAB-ZFP genes acted as repressive cis-elements (Fig. 1b, c). One previous study showed that KAP1, the primary partner of KRAB-ZFPs, was enriched at the 3′ ends of KRAB-ZNF genes, suggesting that KRAB-ZNFs may be recruited to these sites and restrict their expression. In our study, we found that ZNF300 bound to ZNF300ZFR (Fig. 3f, g) and repressed ZNF300 expression by inducing epigenetic modification (Figs. 4, 5). These observations suggests that ZNF300 may repress its own expression. Further UCSC genome browser search showed that ZNF274 might bind to this region. Interestingly, further database analysis revealed that ZNF274 was enriched on its own zinc finger-coding region as well as other KRAB-ZNF zinc finger domain-encoding regions. ZNF263 ChIP-Seq dataset also showed enrichment on zinc finger domain-encoding regions of multiple KRAB-ZFP genes (UCSC genome browser). Therefore, self-inhibition or multilateral inhibition may exist in KRAB-ZFP genes. One can imagine that different KRAB-ZFPs may function in synergy or antagonism. Thus further study on the interaction among KRAB-ZFPs may be important to understand their functions.

Our study also demonstrated that ZNF300 mediated histone modifications in pathological conditions. It is well known that KRAB-ZFPs mediate long-range DNA methylation. However, whether KRAB-ZFPs also mediate histone modifications is not well established, although HDACs are shown to be components in KRAB-ZFP/KAP1 complex. In our study, we showed ZNF300 mediated global change of H3K9Ac and H3K9me3 in K562 cells undergoing megakaryocyte differentiation. Considering that ZNF300 dysregulation contributed to multiple pathological processes, ZNF300 dysregulation may be important to cause or maintain altered histone modifications observed in these pathologies. Further study on how ZNF300 regulates histone modifications may help understand the mechanism leading to altered histone modifications in these pathologies.

In conclusion, our evidence supports that there may exist an unveiled mechanism of ZNF300 self-repression via binding to its own zinc finger domain-encoding region. Our study may put forward new insights into understanding of KRAB-ZFPs.
